# Repetitive transcranial magnetic stimulation impacts the executive function of patients with vascular cognitive impairment: a systematic review and meta-analysis

**DOI:** 10.3389/fneur.2024.1374395

**Published:** 2024-06-19

**Authors:** Xu Wang, Qixin Ding, Yuefang Li, Tianshu Li, Yakun Li, Jialin Yin, Weisheng Zhuang

**Affiliations:** ^1^School of Rehabilitation Medicine, Henan University of Chinese Medicine, Zhengzhou, China; ^2^School of Clinical Medicine, Henan University, Zhengzhou, China; ^3^Department of Rehabilitation Medicine, The First People’s Hospital of Zhengzhou, Zhengzhou, China; ^4^Department of Rehabilitation, Henan Provincial People's Hospital, Zhengzhou, China; ^5^Department of Rehabilitation, School of Rehabilitation Medicine, Henan Provincial People's Hospital, Henan University of Chinese Medicine, Zhengzhou, China

**Keywords:** repetitive transcranial magnetic stimulation, vascular cognitive impairment, executive function, cognition, vascular dementia, meta-analysis

## Abstract

**Objective:**

Executive dysfunction is a core symptom of vascular cognitive impairment (VCI), which seriously affects patients’ prognosis. This paper aims to investigate the effectiveness of rTMS on executive function in VCI.

**Methods:**

The databases selected for this study included Pubmed, Embase, Cochrane Library, China National Knowledge Infrastructure (CNKI), Wanfang, China Science and Technology Journal Database (VIP), and China Biology Medicine Disc (CBM). The screening times were conducted from the time of library construction until August 23, 2023. The inclusion criteria for this meta-analysis were randomized controlled trials (RCTs) on rTMS for VCI, which include executive function scores. The primary metrics were executive subscale scores of the Cognitive Comprehensive Scale and total scores of the Executive Specificity Scale. The secondary metrics were subscale scores of the Executive Specificity Scale. The quality of each eligible study was assessed using the Cochrane Risk of Bias tool. Meta-analysis and bias analysis were performed using Stata (version 16.0) and RevMan (version 5.3).

**Results:**

A total of 20 high-quality clinical RCTs with 1,049 samples were included in this paper. The findings from the primary outcomes revealed that within the rTMS group, there were significantly higher scores observed for the executive sub-item on the cognitive composite scale (SMD = 0.93, 95% CI = 0.77–1.08, *p* < 0.00001, *I*^2^ = 14%) and the total score on the executive specific scale (SMD = 0.69, 95% CI = 0.44–0.94, *p* < 0.00001, *I*^2^ = 0%) compared to the control group. As for the secondary outcome measures, as shown by the Trail Making Test-A (time) (MD = −35.75, 95% CI = −68.37 to −3.12, *p* = 0.03, *I*^2^ = 55%), the Stroop-C card (time) (SMD = −0.46, 95% CI = −0.86 to −0.06, *p* = 0.02, *I*^2^ = 0%) and the Stroop-C card (correct number) (SMD = 0.49, 95% CI = 0.04–0.94, *p* = 0.03, *I*^2^ = 0%), the experimental group shorts time and enhances accuracy of executive task in comparison to the control group. Subgroup analysis of the main outcome demonstrated that intermittent theta burst stimulation (iTBS), higher frequency, lower intensity, longer duration, and combined comprehensive therapy exhibited superior efficacy.

**Conclusion:**

rTMS is effective in the treatment of the executive function of VCI. The present study has some limitations, so multi-center, large-sample, objective indicators and parameters are needed to further explore in the future.

**Systematic review registration:**https://www.crd.york.ac.uk/prospero/, CRD42023459669.

## 1 Introduction

Owing to the aging of the population, the number of patients with cognitive impairment is increasing year by year, and it is predicted that 81.1 million people will be affected by 2040. The trend increases the social and economic burden, which has become the public challenge of the times (1). Among the various causes of cognitive impairment, cerebrovascular disease stands out as the second, particularly in East Asia (2). Vascular cognitive impairment (VCI) refers to cognitive decline due to brain vessel damage, and it ranges from mild vascular cognitive impairment to vascular dementia (VaD) (3). Its symptoms mainly include four cognitive areas, among which executive dysfunction is its core symptom. Studies have also demonstrated that prefrontal subcortical function impairment is a prevalent neurophysiology of VCI, which significantly impairs executive function and negatively impacts the patient’s quality of life (2, 4, 5). At present, VCI is mainly treated by prevention and drugs. However, simple drug treatment is usually not effective and there is a lack of specific treatment. More and more scholars have begun to pay attention to its non-drug treatment, among which the commonest technologies for cognitive enhancement of stroke patients include computer cognitive training, virtual reality, non-invasive brain stimulation, and brain-computer interface (6).

Transcranial magnetic stimulation (TMS) is a non-invasive regulatory technique. Its mechanism entails the application of pulsed magnetic fields generated by a coil to selectively enhance or suppress the excitability of the cerebral cortex. This alters neuronal activity and modulates the functional connectivity within the brain network, thereby influencing cognitive and executive functions (7, 8). Most cognitive studies have used the dorsolateral prefrontal cortex (DLPFC) as a stimulating region, which is mainly related to executive and attention functions. In recent years, the therapeutic effects of rTMS on executive function have been investigated, and the possible mechanisms are related to increasing synaptic connections, promoting neuroplasticity, accelerating cerebral blood flow, facilitating neuronal cell growth, and improving local brain metabolism (9). Several studies have shown the potential of TMS in the recovery of executive function of diverse neuropsychiatric diseases. Moser et al. (10) found significant improvements in TMT-B tests in middle-aged and elderly patients with refractory depression after rTMS stimulation; Zheng et al. (11) found that improved executive functioning was associated with biochemical changes in cingulate neurochemistry after high-frequency rTMS in young depressed patients; Cricstancho et al. (12) obtained similarly beneficial results after stimulation of iTBS in depressed patients. Herremans et al. (13) treated alcohol-dependent patients with high-frequency rTMS and found that it stabilized cognitive performance in an executive control task; Ameis et al. (14) found improved executive functioning after rTMS in participants with autism who had more severe deficits in adaptive functioning. In particular, since Rektorova et al. (15) revealed the beneficial effects of high-frequency rTMS on executive function in a blinded crossover trial involving seven patients with post-stroke executive dysfunction, more researchers have begun to focus on the effects of rTMS on executive function with VCI. However, it is worth noting that existing these trials are generally constrained by limited sample sizes, geographical variations, and other factors, leading to non-uniform quality levels. Consequently, further research is imperative to validate these findings. According to the search, existing meta-analyses (16–20) have focused on rTMS for the treatment of cognitive impairment in stroke, with no meta-analysis of rTMS for vascular executive dysfunction now. Therefore, this paper aims to (1) demonstrate the effectiveness of rTMS for the executive function of patients with VCI and (2) further subgroup analyses to assess the effects of multiple factors on the treatment.

## 2 Data and methods

### 2.1 Search strategy

The search time is from the establishment of the database to August 23, 2023, regardless of language, location, and other factors. The search databases included Pubmed, Embase, Cochrane Library, CBM, CNKI, and Wanfang. The search terms include transcranial magnetic stimulation, cognitive disorder, executive function, etc.

### 2.2 Inclusion and exclusion criteria

Inclusion criteria: (1) Randomized controlled trial (RCT); (2) the intervention method of the experimental group was transcranial magnetic stimulation combined with basic treatment, and the intervention method of the control group was non or sham-stimulation combined with the same basic treatment; (3) patients with VCI were treated; (4) outcome indicators include executive function scores. Exclusion criteria: (1) insufficient data; (2) publications; (3) the literature with original data still cannot be found after trying all methods.

### 2.3 Outcome indicators

There are two outcome indicators to evaluate executive function: one is a screening scale including executive function score, such as the Montreal Cognitive Assessment (MOCA), Mini-mental State Examination (MMSE), the Oxford Cognitive Screen (OCS), etc.; and the other is a specific scale to assess executive function, such as Frontal Assessment Battery (FAB), Trail Making Test (TMT), Stroop test, clock-drawing experiment (CDT), Behavioral Dysexecutive Syndrome (BADS), Tower of London test (TOL), etc. The primary indicators in this paper are the executive scores of the composite scale (designated as Indicator 1) and the total score of the specificity scale (designated as Indicator 2). Additionally, the secondary outcomes were the sub-scores of the specificity scale.

### 2.4 Literature screening, data extraction, and bias risk assessment

The study was collaboratively conducted by three authors. Two researchers independently screen, data extraction, and bias analysis. In case of disagreements, the third author was responsible for facilitating joint discussions. During the initial screening, we focused on the title and abstract. Subsequently, during the second screening, we read the original text to determine its compliance with the inclusion and exclusion criteria. The extracted data included: (1) basic information of the literature: author, year, etc. (2) subjects: disease type, group, number of cases, etc. (3) intervention methods: stimulating site, frequency, intensity, basic treatment, etc. (4) Outcome indicators: time, scale, data (5) risk of bias results. The risk of bias was assessed according to the Cochrane risk assessment tool (21). This involved seven different types of biases: attrition bias (incomplete outcome data), selection bias (unbiased sequence generation and allocation concealment), reporting bias (selective result reporting), blinding bias (unbiased performance and detection), and other biases. The risk of bias for each element was categorized as low, unknown, or high.

### 2.5 Statistical analysis

The results of the meta-analysis were processed by Revman 5.3 software (version 5.3; Cochrane Collaboration, Software Update, Oxford, UK). The continuous variables of the same indicator are compared with MD and 95% CI, and different indicators evaluating the same function can be treated with SMD and 95% CI (22). The heterogeneity of results was determined by *I*^2^: (1) The fixed-effect model was selected when *I*^2^ < 50%, and the random effect model was selected when *I*^2^ > 50%;(2) When *I*^2^ > 75%, it indicates that the heterogeneity is too high, and sensitivity analysis or subgroup analysis is needed to find the cause. If the heterogeneity cannot be reduced, a system description is performed. Stata software (version 16.0^1^) was used to make funnel plots to evaluate publication bias. Finally, GRADE profiler software was used to evaluate the quality of the meta-analysis results.

## 3 Results of meta-analysis

### 3.1 Screening results and methodological quality evaluation results

**Figure 1:**
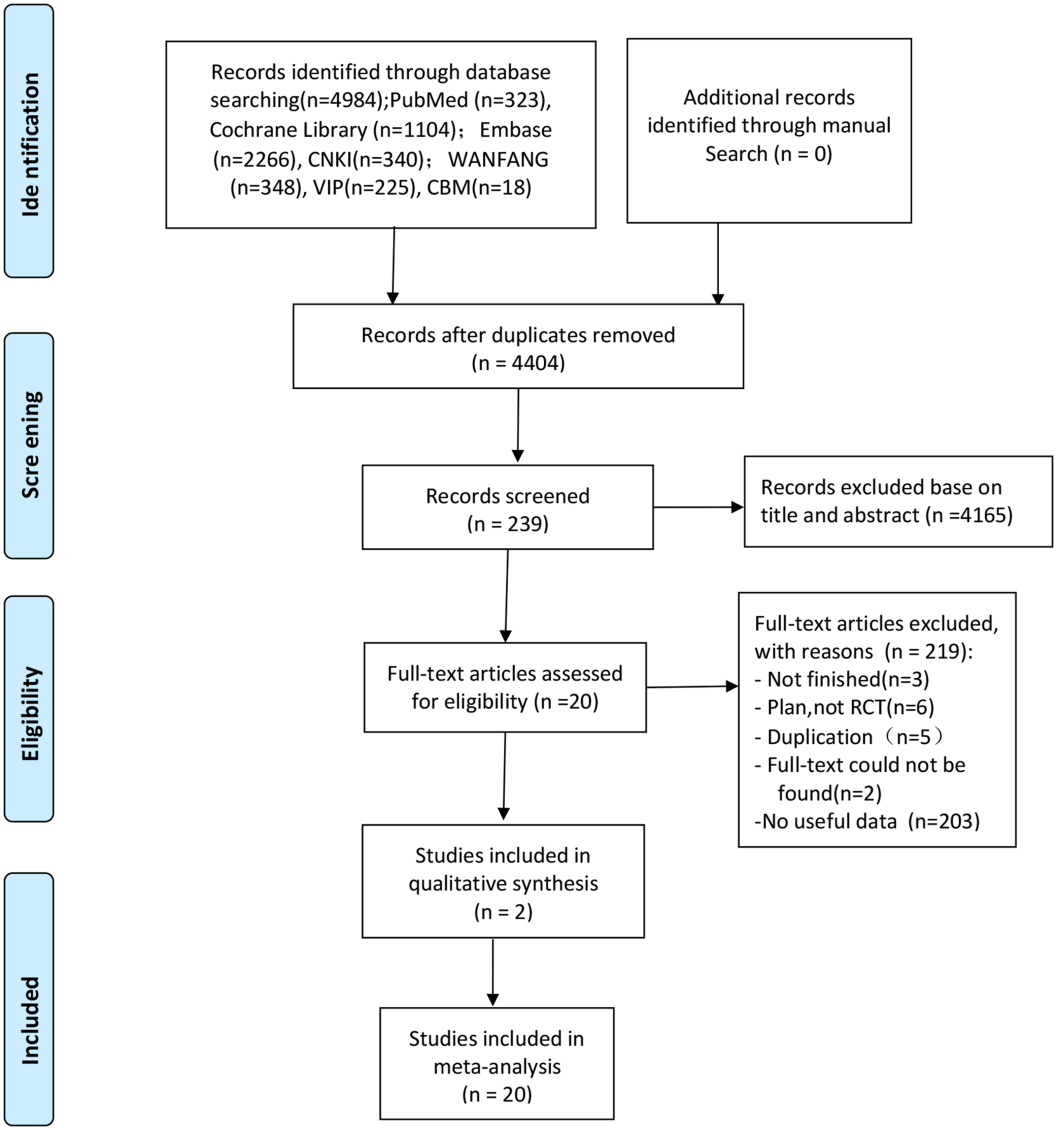
The flowchart of the literature search and screening process.

**Figure 2:**
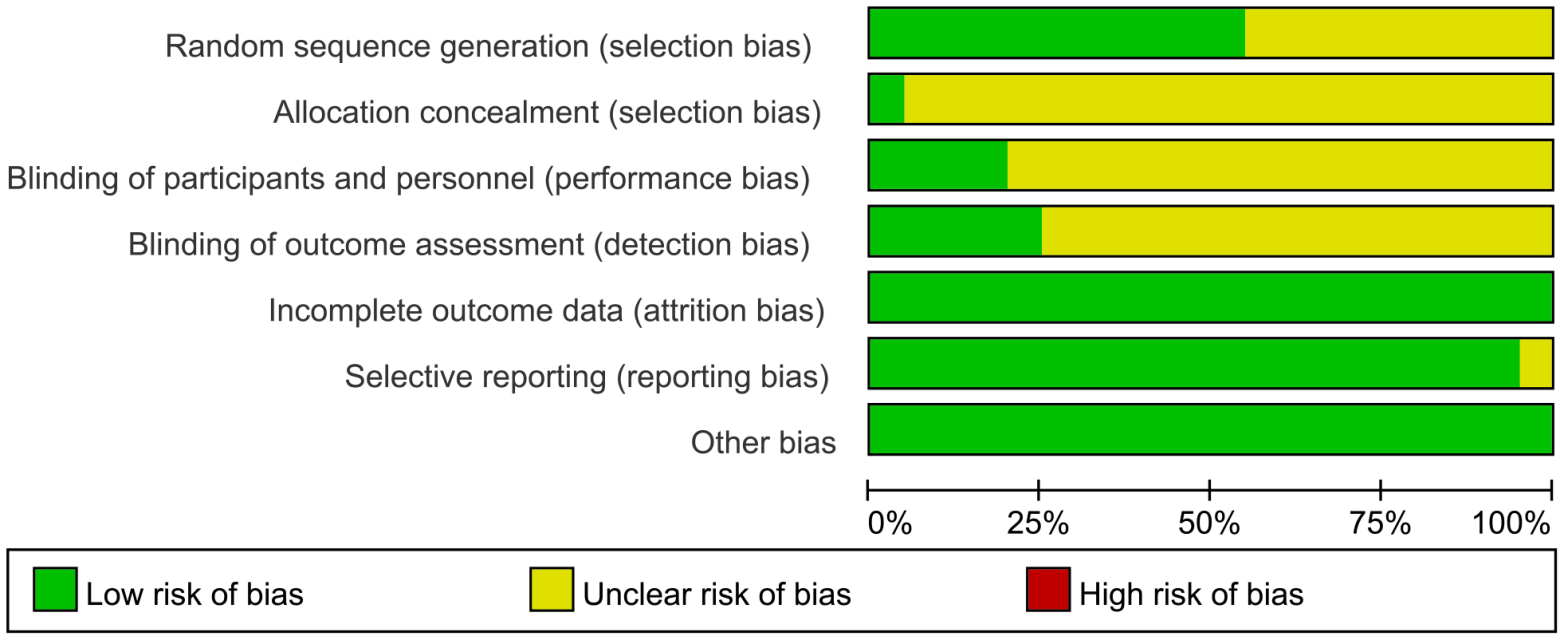
Risk of bias graph: review authors’ judgments about each risk of bias item presented as percentages across all included studies.

**Figure 3:**
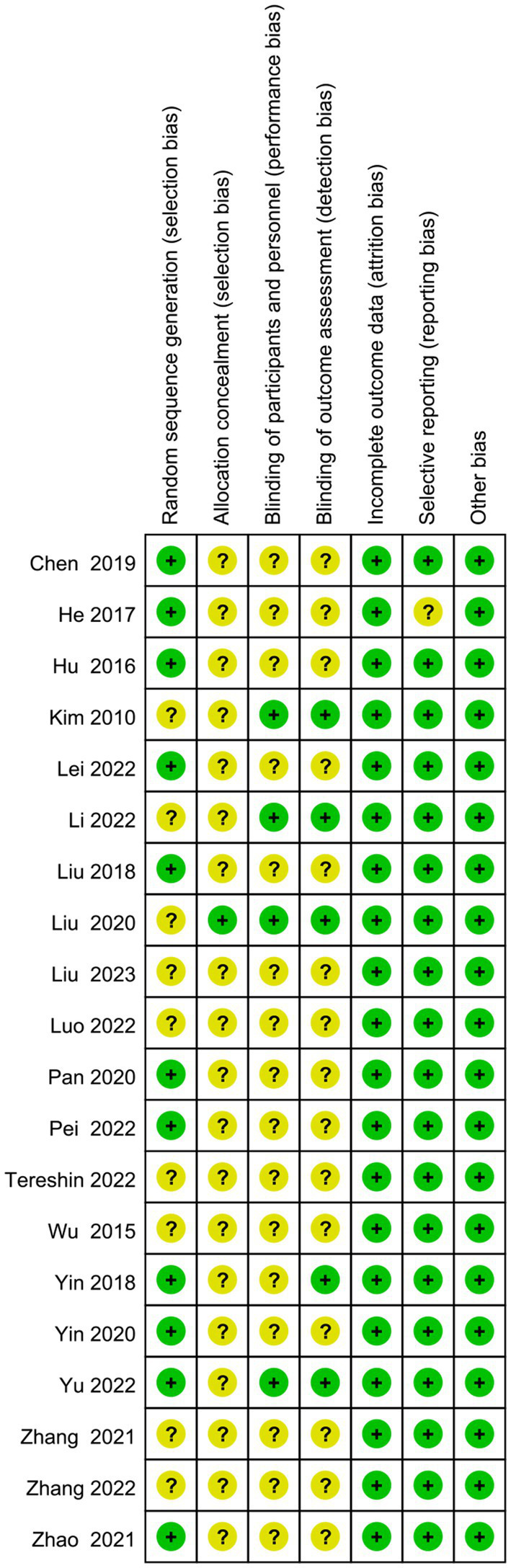
Risk of bias summary: review authors’ judgments about each risk of bias item for each included study.

The screening flow chart is shown in Figure 1. A total of 4,984 articles were retrieved based on the search strategy. After reading the abstract and title, 4,165 articles were excluded, leaving 239 articles for preliminary screening. Finally, after a thorough examination of the full text, 20 articles were included, encompassing a total of 1,049 patients. The risk of bias assessment (Figures 2, 3) unveiled that out of the 20 articles, 11 explicitly described the randomization procedure, while 9 only mentioned randomization without specifying the method. Additionally, 4 articles implemented double-blind, 1 employed a single-blind approach, while the rest did not mention their blinding strategy. Only 1 article among the 20 used allocation concealment. At present, no other risks have been identified. In conclusion, none of the articles manifested high-risk indicators.

### 3.2 Basic features of included studies

The basic characteristics of the 20 literatures are shown in Table 1. Regarding the disease type, 16 articles centered on patients suffering from poststroke cognitive dysfunction. Two articles explored cognitive impairment resulting from small vessel disease, and one article investigated vascular cognitive impairment. Concerning the control group, 10 articles were subjected to sham stimulation, while another 10 articles did not receive any stimulation. Of the stimulus plan, 12 underwent rTMS, coupled with medication and rehabilitation. Eight articles underwent rTMS, along with either medication or rehabilitation. The stimulation intensity ranged from 80 to 100% of the motor threshold (MT), and only one article utilized low-frequency (1 Hz) stimulation. All other articles employed high-frequency stimulation (5 Hz or 10 Hz). Regarding outcomes, 10 articles integrated screening scales that assessed executive function through subitem scores, while 12 articles employed specific scales to evaluate executive function.

**Table 1:** Basic information of the included studies.

Author/year	Disease	*n* (T/C)	Stimulation (T/C)	Basic treatment	Stimulation side	Parameter	Time	Outcomes
Liu 2023	PSCI	31/29	rTMS/No	3, 4	Left DLPFC	5 Hz 80%MT; 2,000 pulses	1 time/day; 3w	MOCA
Zhang 2022	PSCI	20/20	rTMS/No	3	Left DLPFC	5 HZ 80%MT; 3,000 pulses	5 times/w; 4w	SCWT
Luo 2022	PSCI	30/30	rTMS/No	3, tDCS	Left DLPFC	5 HZ 80%MT	5 times/w; 8w	TMT, SCWT
Pei 2022	PSCI	31/29	iTBS/sham	3 (computer)	Left DLPFC	5 Hz 80%MT; 1,200 pulses	5 times/w; 4w	FAB
Lei 2022	PSCI	31/29	rTMS/No	3, 4	Left DLPFC	10 Hz, 80%MT	5 times/w; 8w	MOCA
Yu 2022	PSEI	9/9	rTMS/sham	3, 4	Left DLPFC	5 Hz 80%MT; 1,200 pulses	5 times/w; 2w	SCWT
Tereshin 2022	PSCI	9/18	rTMS/No	2	Left DLPFC	10 HZ; low intensity	3w	FAB
Li 2022	PSCI	28/30	iTBS/sham	2	Left DLPFC	5 Hz 100%MT; 600pulses	5 times/w; 2w	OCS
Zhang 2021	PSCI	32/34	rTMS/No	1	Left DLPFC	10 Hz, 100%MT	12w	MOCA
Zhao 2021	PSCI	31/35	rTMS/No	2	Left DLPFC	10 Hz 80%MT; 1,200pulses	5 times/w; 4w	BADS
Liu 2020	PSCI	29/29	rTMS/sham	2	Left DLPFC	10 Hz 90%MT; 700 pulses	5 times/w; 4w	TMT-A
Yin 2020	PSCI	16/18	rTMS/sham	3 (computer)	Left DLPFC	10 Hz 80%MT; 2,000pulses	5 times/w; 4w	MOCA, VST
Pan 2020	VCI	53/53	rTMS/sham	2	Left DLPFC	10 Hz 100%MT; 3,000pulses	5 times/w; 4w	MOCA, CDT
Chen 2019	PSCI	70/70	rTMS/No	3	Left DLPFC	10 Hz,80%MT	5 times/w; 4w	MOCA
Yin 2018	PSCI	12/13	rTMS/sham	3 (computer)	Left DLPFC	10 Hz 80%; 2,000pulses	5 times/w; 4w	VST
Liu 2018	PSEI	18/18	rTMS/sham	3	Left DLPFC	10 Hz, 90%MT; 740 pulses	5 times/w; 2w	WCST SCWT TMT-A
He 2017	VCIND	16/14	rTMS/No	3	Left DLPFC	10 Hz, 80–120%MT	5 times/w; 4w	MOCA
Hu 2016	VCIND	30/30	rTMS/No	4	Left DLPFC	10 Hz 100%MT; 3,000 pulses	5 times/w; 4w	MOCA
Wu 2015	VCIND	18/15	rTMS/No	1	Healthy side	1 Hz, 80–120%MT; 600–800 pulses	5w	MOCA
Kim 2010	PSCI	6/6	rTMS/sham	No	Left DLPFC	10 Hz 80%MT; 450 pulses	5 times/w; 2w	TOL

rTMS, repetitive transcranial magnetic stimulation; iTBS, intermittent theta burst stimulation; PSCI, post stroke cognitive impairment; PSEI, post stroke executive impairment; VCIND, Non-dementia vascular cognitive impairment; VCI, vascular cognitive impairment; 1, drugs and rehabilitation therapy; 2, dugs; 3, rehabilitation therapy; 4, acupuncture therapy; DLPFC, dorsolateral prefrontal cortex; MT, motor threshold; MOCA, Montreal Cognitive Assessment; MMSE, Mini-mental State Examination; OCS, the Oxford Cognitive Screen; TMT, Trail Making Test; FAB, Frontal Assessment Batter; SCWT, Stroop Color-word Test; VST, Victoria Stroop test; WCST, Wsiconsin card sorting test; CDT, clock-drawing experiment; BADS, Behavioral Dysexecutive Syndrome; TOL, Tower of London test.

### 3.3 Results of a meta-analysis

#### 3.3.1 Key indicator: executive subitems of the generality scale (indicator 1)

**Figure 4:**
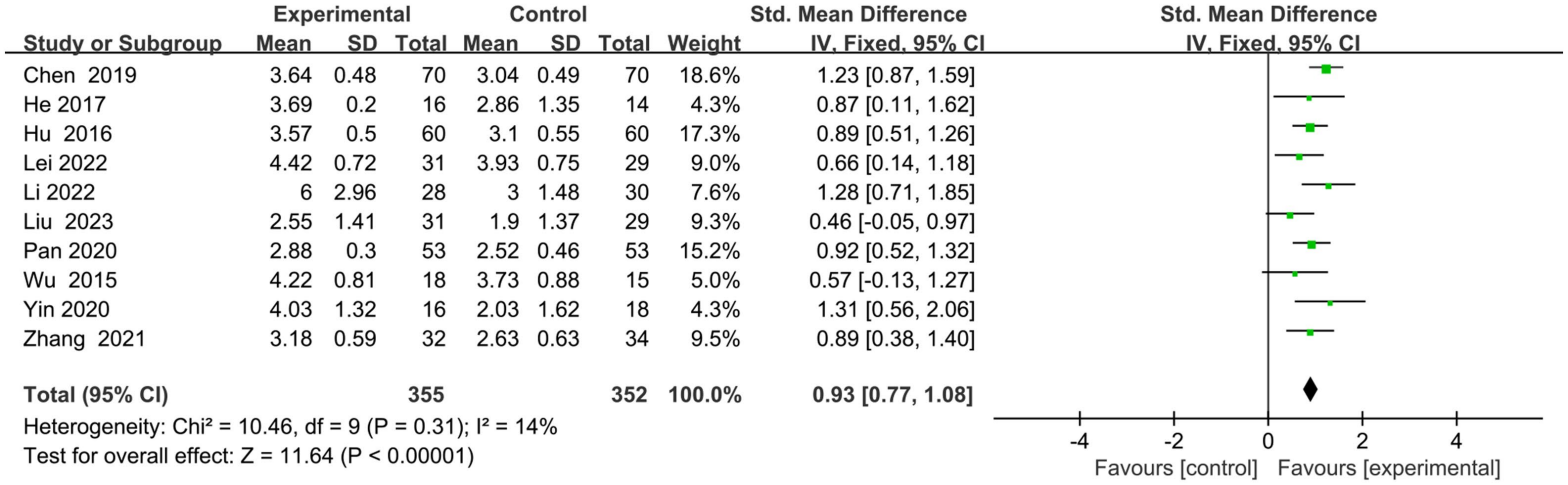
Indicator 1: Frost plot of the executive subscale scores of the Cognitive Comprehensive Scale.

Ten articles (23–32) had executive function scores on the screening scales (Figure 4). Eight (24–28, 30–32) used MOCA-visual–spatial and executive scales, one (29) used MOCA-executive scales, and one (23) employed OCS-executive scales. Following a comprehensive analysis with an *I*^2^ value of 14%, a fixed-effects model was employed. The results indicated that the experimental group exhibited superior enhancements in executive function compared to the control group (SMD = 0.93, 95% CI = 0.77–1.08, *p* < 0.00001).

#### 3.3.2 Key indicator: specificity scale for executive functioning (indicator 2)

**Figure 5:**
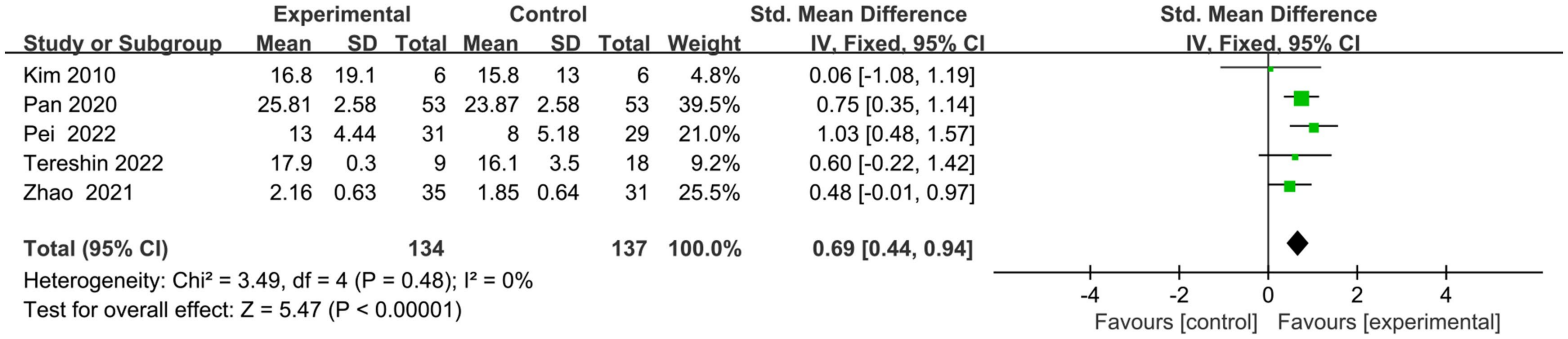
Indicator 2: Frost plot of the total scores of the Executive Specificity Scale.

Five articles (24, 33–36) employed specialized scales to evaluate executive performance (Figure 5). Out of these, two (34, 35) utilized FAB, one (33) utilized the TOL, another (24) involved the CDT, and the last one (36) utilized the BADS. The combined analysis using a random effects model yielded an *I*^2^ value of 0%. The findings revealed a significant enhancement in executive function among the transcranial magnetic stimulation group in contrast to the control group (SMD = 0.69, 95% CI = 0.44–0.94, *p* < 0.00001).

#### 3.3.3 Sub-indicators: sub-items of the specificity indicator

**Figure 6:**
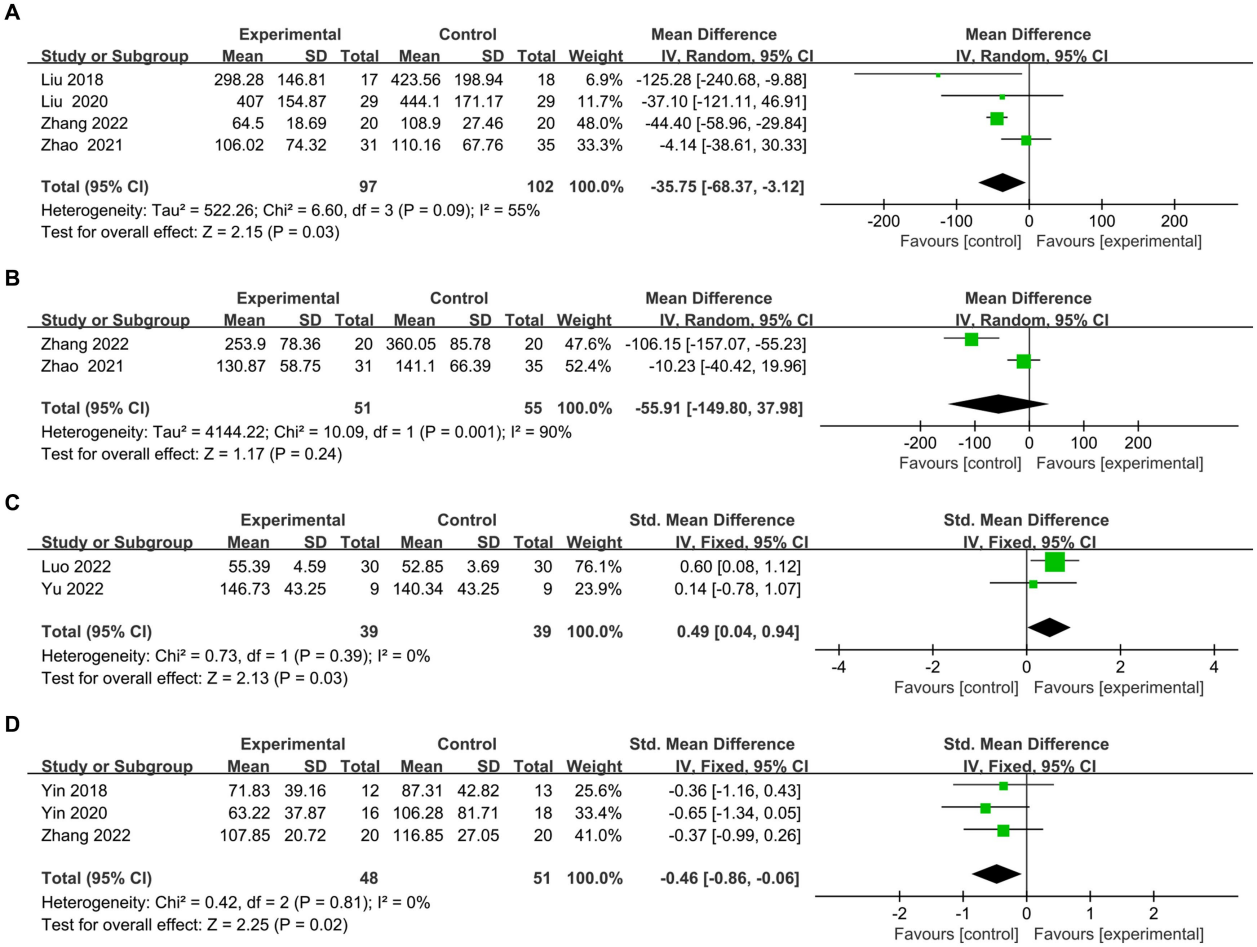
Frost plot of subscale scores of the Executive Specificity Scale. **(A)** Frost plot of TMT-A. **(B)** Frost plot of TMT-B. **(C)** Frost plot of the Stroop-C(n). **(D)** Frost plot of Stroop-C(t). TMT-A, Trail Making Test-A test; TMT-B, Trail Making Test-B test; Stroop-C(n), correction number of the C card of the Stroop test; Stroop-C(n), time of the C card of the Stroop test.

Here we discuss the subitems of two scales (TMT test and Stroop test). (1) The TMT is one of the most commonly used scales for assessing executive function. The TMT-A represents the right cerebral hemisphere’s function, indicating rapid visual search, visuospatial ordering, and perceptual-motor speed abilities. The TMT-B represents the left hemisphere’s function, indicating overall visuospatial scanning, perceptual-motor speed, and stereotypical switching abilities. Four articles (26, 36–39) evaluated the time on TMT-A, and meta results showed in Figure 6A that the rTMS group spent less time on A test (MD = −35.75, 95% CI = −68.37 to −3.12, *I*^2^ = 55%, *p* = 0.03). Two papers (36, 39) evaluated the time of TMT-B. However, owing to high heterogeneity (I2 = 90%) (Figure 6B), both Zhang et al. (39) (MD = −106.15, 95% CI = −157.07 to −55.23) and Zhao et al. (36) (MD = −10.23.95% CI = −40.42 to 19.96)’s experimental group took less time than the control group for qualitative analysis. However, owing to significant heterogeneity (*I*^2^ = 90%) (Figure 6B), both Jingjing et al. (39) (MD = −106.15, 95% CI = −157.07 to −55.23) and Jingjing et al. (39) (MD = −10.23.95% CI = −40.42 to 19.96)’s experimental group took less time than the control group for qualitative analysis. (2) There exist various versions of the Stroop test, such as the Symbol Color Word Test (SCWT) and the Visual Stroop Test (VST), which primarily assesses the ability of the execution function to resist interference. Two articles (40, 41) evaluated the correct number of Stroop scale, and the combined analysis found that the correction of the experimental group was better than the control group (SMD =0.49, 95% CI = 0.04 to 0.94, *I*^2^ = 0%, *p* = 0.03) (Figure 6C). Three articles (25, 39, 42) assessed the time to complete card *C. meta*-analysis showed that the rTMS group took less time than the control group (SMD = −0.46.95% CI = −0.86 to −0.06, *I*^2^ = 0%, *p* = 0.02) (Figure 6D).

#### 3.3.4 Subgroup analysis results

Table 2 shows the results of the subgroup analysis of the main indicators from all aspects. The results are as follows: (1) The improvement of rTMS in combination with comprehensive therapy (Indicator 1: SMD, 95% CI = 0.92, 0.72–1.12; Indicator 2: SMD, 95% CI = 1.03, 0.48–1.57) is superior to that in combination with monotherapy (Indicator 1: SMD, 95% CI =0.85, 0.56–1.14; Indicator 2: SMD, 95% CI = 0.60, 0.32–0.88); (2) the effect of intervention time for ≥4 w (Indicator 1: SMD, 95% CI = 0.95, 0.78–1.12; Indicator 2: SMD, 95% CI = 0.74, 0.47–1.00) was superior to that for <4 w (Indicator 1: *p* = 0.08; Indicator 2: *p* = 0.22). (3) The application of intermittent theta burst stimulation (iTBS) (SMD, 95% CI = 1.28, 0.71–1.85), a novel therapeutic modality of rTMS, may be superior to traditional rTMS (SMD, 95% CI = 0.90, 0.74–1.06) in improving executive function. (4) Higher frequencies (10 Hz) (*p* < 0.0001) may demonstrate greater efficacy in executing functions when compared to both low frequency (*p* = 0.11) and high frequencies of 5 Hz (*p* = 0.08). (5) Lower-intensity (80%) rTMS (SMD, 95% CI = 0.90, 0.49–1.32) may be better than higher-intensity (SMD, 95% CI = 0.87.0.65–1.08) for improving executive functioning. Overall, it is possible that a combination of comprehensive therapy, intervention for a longer period (>4w), higher frequency (10 Hz), lower intensity (80%), and a specific type (iTBS), may have a better effect on improving the executive function of VCI.

**Table 2:** Subgroup analysis of key indicators.

Indicator	Subgroup		*n*	SMD	95% CI	*p*	*I* ^2^
Indicator 1	Combination treatment	Comprehensive therapy	6	0.92	0.72, 1.12	<0.00001	37%
		Monotherapy	3	0.85	0.56, 1.14	<0.00001	0%
	Time	<4w	1	0.46	−0.05, 0.97	0.08	
		≥4w	8	0.95	0.78, 1.12	<0.00001	0%
	Type	iTBS	1	1.28	0.71, 1.85	<0.0001	
		rTMS	9	0.9	0.74, 1.06	<0.00001	10%
	Frequency	Low (1 Hz)	1	0.57	−0.13, 1.27	0.11	
		High (5 Hz)	1	0.46	−0.05, 0.97	0.08	
		High (10 Hz)	7	0.97	0.80, 1.15	<0.00001	0%
	Intensity	80%	4	0.9	0.49, 1.32	<0.00001	62%
		>80%	5	0.87	0.65, 1.08	<0.00001	0%
Indicator 2	Combination treatment	Comprehensive therapy	4	1.03	0.48, 1.57	0.0002	0%
		Monotherapy	1	0.6	0.32, 0.88	<0.0001	
	Time	<4w	2	0.42	−0.25, 1.08	=0.22	0%
		≥4w	3	0.74	0.47, 1.00	<0.00001	6%

### 3.4 Publication bias and grade quality evaluation

**Figure 7:**
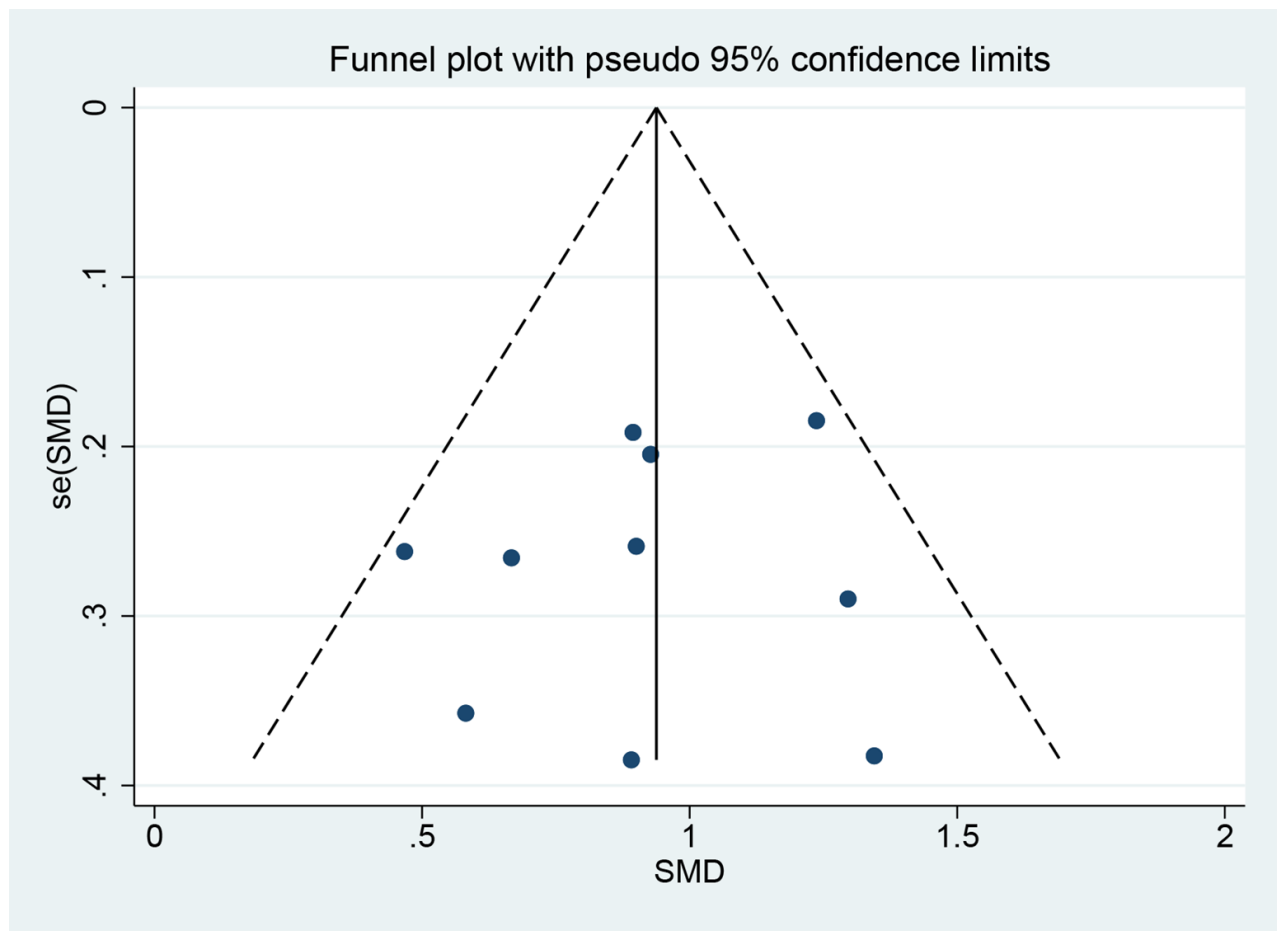
Funnel plots of a key indicator of executive function.

To evaluate publication bias, we conducted an analysis using funnel plots (Figure 7) and the Egger test. The results demonstrated a symmetrical distribution on both sides of the funnel plots and a *p*-value of 0.635 on the Egger test, indicating the absence of publication bias. Furthermore, a sensitivity analysis was carried out for each indicator, and the results consistently demonstrated stability. Ultimately, the quality assessment of the meta-analysis conducted in this study employed the GRADE approach. The evaluation revealed that our primary outcomes, namely the screening scale sub-score (indicator 1) and specificity scale total score (indicator 2), exhibited high quality. Meanwhile, the remaining outcomes, such as TMT-A and Stroop scales (time and correction number), displayed a moderate quality.

## 4 Discussion

Executive function is a high-level cognitive function with core components of abstract thinking, working memory, stereotype transfer, and reflective inhibition (43, 44). Executive dysfunction is the core symptom of VCI, which seriously affects the prognosis of stroke and poses a significant clinical challenge. Scholars are increasingly focused on this issue, highlighting the critical need to find an effective remedy (45). Studies have found that rTMS shows great potential in the treatment of executive function in VCI. The pulsed magnetic field of rTMS influences brain tissues (46), affecting synaptic structure (47–49), neurotransmitters (50, 51), brain-derived trophic factors (50, 52, 53), regulating neuronal excitatory gene expression (52, 54, 55), and altering neuronal membrane potentials (56, 57). The neurobiological mechanisms underlying the cognitive-enhancing effects of rTMS are not fully understood. The primary mechanism involves cortical plasticity, specifically the change of long-term potentiation (LTP) and long-term depression (LTD) (58–60). Stroke recovery relies on two competing neuroplastic remodeling mechanisms: interhemispheric competition and compensation. rTMS can enhance excitability in the affected hemisphere or modulate activity in the unaffected hemisphere (61). Animal studies employing rTMS for VCl have revealed changes in hippocampal synaptic morphologies, increased LTD induction, and heightened synaptic plasticity (52, 56, 62). The second manifestation involves an impact on the cholinergic system (63, 64). Acetylcholine (Ach) serves as an excitatory neurotransmitter crucial for learning and memory functions. Zhang et al. (50) applied rTMS (0.5 Hz, 1.33 T) to rats with vascular dementia (VD) over 30 days. They noted a substantial rise in the activities of acetylcholinesterase (AchE) and choline acetyltransferase (ChAT), an augmentation in neuronal cholinergic density, and a notable amelioration of learning and memory impairments in VD rats. Furthermore, apoptosis stands as the predominant cause of neuronal demise, notably in ischemic regions. Animal studies have demonstrated that rTMS exhibits anti-apoptotic and neuroprotective properties. By stimulating the PI3K/Akt/mTOR pathway, rTMS hinders apoptosis in hippocampal neurons (53). Guo et al. (65) identified significantly elevated levels of BDNF, TrkB, and the anti-apoptotic protein Bcl-2 in the ischemic hippocampus. In addition, astrocytes possess the capacity to shield brain tissue and mitigate disability stemming from ischemic brain disorders. rTMS (10 Hz) can enhance cognitive function by mitigating white matter lesions, reducing pro-inflammatory cytokine levels, elevating anti-inflammatory cytokine levels, and inducing the transformation of microglial cells into the M2 phenotype (66). These process influences the activity and functional connectivity of specific brain regions, potentially serving as one of the mechanisms to regulate functional brain activity and enhance executive function.

At present, rTMS is still being explored for its therapeutic applicability in the executive function of VCI. While numerous RCTs have been conducted, a comprehensive meta-analysis on this topic is yet to be established. Based on this, this study aims to fill this research gap by incorporating a total of 20 RCTs that investigate the efficacy of rTMS in improving executive function following VCI. The conclusions of the meta-analysis and systematic review are as follows (1) rTMS can improve the executive function of VCI; (2) Subgroup analyses showed that higher frequency, lower intensity, iTBS, combined comprehensive therapy, and longer interventions yielded better gains. The meta-results are consistent with the meta-analysis results of Gao et al. (18) and Han et al. (20). However, unlike these studies, they only incorporated two articles as part of their cognitive outcome analysis without conducting a comprehensive analysis. This paper addresses this limitation by providing more extensive evidence with 20 RCTs and full indicators. Alongside the inclusion of the 20 RCTs, it is noteworthy to mention that Rectorova et al. (15) conducted a well-designed crossover, sham-controlled trial in 2005. This study focused on the application of rTMS for improving executive impairment following stroke (referred to as PSEI). The results indicated that stimulating the left DLPFC led to notable improvements in patients’ problem-solving ability, as assessed by the TMT. However, the efficacy in terms of memory function and verbal fluency was found to be insignificant. In 2008, Sedlackva et al. (67) expanded their study to include the MCl-V of seven patients as a before-and-after control trial, confirming that rTMS had an equivalent effect on this subgroup. Additionally, Aref (68) conducted a study involving 40 patients to investigate the efficacy of high-frequency rTMS in treating cerebral small-vessel disease. The results from their RCT indicated that the FAB scores of the true-stimulation group were significantly higher than those of the sham-stimulation group. However, the findings were not incorporated into the present meta-analysis due to the lack of post-treatment data. Furthermore, Yuanwen et al. (38) conducted another RCT, concluding that rTMS treatment for PSEI led to improved WCST scores. Nonetheless, this study was excluded from the meta-analysis due to inconsistencies in comparison to other RCTs included in this meta-analysis. With the help of functional near-infrared spectroscopy (fNIRS), a recent study by Liu et al. (69) observed individuals with post-stroke executive impairment following rTMS activation of the left DLPFC. The researchers discovered that the time and number of error scores of the Stroop test were significantly higher in these patients, which correlated with the enhanced functional connectivity between various brain regions. Notably, this activation involved the left DLPFC, the right PMC, and the right SM1 brain regions. These trials all further support the conclusions of this article.

The potential of rTMS in treating executive function after VCI is immense, and as the evidence for its effectiveness grows, the need for further optimization becomes increasingly crucial. Currently, there is no uniform clinical standard for the treatment mode. The most common stimulation site for rTMS is DLPFC, with an intensity of 80% ~ 120% motor threshold (MT), a frequency of 5 ~ 25 Hz, and time intervals of 20 ~ 30 s. The treatment duration typically extends 3 weeks or longer (70). Numerous factors, including stimulation site, combined modalities, intervention timing, and stimulation parameters, influence the therapeutic outcomes of rTMS. Hence, this paper focuses on subgroup analysis to deepen the primary findings. In terms of stimulation sites, it is known that functional changes in the DLPFC can lead to cognitive and executive deficits (71). Since the patient is right-handed, the current RCT primarily targets the left DLPFC, with only one article in this paper examining the healthy DLPFC, which reduces heterogeneity. In terms of the combination modality of rTMS, subgroup analyses showed that rTMS combined with comprehensive therapy was superior to combined with monotherapy, suggesting that rTMS can add to cognitive routine treatment. Previous studies (72, 73) have also shown that rTMS in combination with other therapies for treating executive dysfunction is a significant trend for the future. In terms of intervention time, the results for both primary indicators 1 and 2 showed better improvement in executive functioning of ≥4 weeks, probably due to the cumulative effect of stimuli. Regarding stimulation type, iTBS is a unique type of rTMS characterized by a 50 Hz pulse with 3 pulses administered every 200 ms (7). Previous studies have confirmed that iTBS has superior effects on cognitive function than conventional rTMS (60, 61), which is consistent with the results of the subgroup analyses in this paper. Concerning stimulation frequency, current clinical research results mainly focus on high-frequency rTMS (10 Hz) to treat the executive function of VCI. 5 Hz high frequency or 1 Hz low frequency may not be effective, but numerous studies have found that low-frequency rTMS can improve cognitive function and enhance neuroplasticity (45). This paper has fewer articles on lower frequencies (one at 5 Hz, one at 1 Hz). Due to the limitations of sample size, their effectiveness needs further verification through original experiments. Regarding stimulation intensity, subgroup analyses showed that lower intensities (80%MT) may be more effective. Zhang et al. (74) explored prefrontal optimal intensity with the fNLRS and found that intensity was nonlinear with prefrontal cortical blood flow and that a 70% MT effect may be better than a 100% effect. However, the interactions between the parameters are not yet clear and need to be further explored in the future.

The therapeutic efficacy of rTMS is influenced by a variety of factors, such as age, sex, handedness, infarct area and size, cardiovascular risk factors, and education level (73). Age is an independent risk factor for executive dysfunction. The results of the study by Yaning et al. (75) showed that, in comparison to younger age groups, showed that, in comparison to younger age groups, older adults had poorer executive scores. Additionally, Mally et al. (76) reported executive function declines and rTMS therapy effects wear off faster in individuals 65 and up. Gender-specific prognoses have been observed in VCI (77). Cantone et al. (78) found that men with mild VCI had worse cognitive and functional status compared with women. This study is the first to emphasize gender-specific alterations in intracortical and corticospinal excitability following rTMS. Executive function primarily stimulates left DLPFC, as confirmed by related research. However, 21.4% of right-handed individuals have their DLPFC located mainly in the right hemisphere rather than the presumed left hemisphere, and 16.7% of left-handed individuals have their DLPFC located mainly in the left hemisphere (79). This shows that a scanner for localizing the potential DLPFC for precise stimulation also significantly impacts prognosis. Brain regions associated with executive regions include the frontal-striatal loops and the cerebellum, and injuries to these regions result in more severe impairments in executive function, which can affect the therapeutic efficacy of rTMS (80). There are no original studies on these relevant factors, and further exploration is necessary. Currently, RCT stimulation sites are mostly limited to the DLPFC, but stimulating the M1 area has been demonstrated to effectively enhance cognitive capabilities. Experiments involving rTMS stimulation of the bilateral DLPFC have yielded favorable outcomes (81). Diseases are characterized by damage to multiple networks and stimulation of other regions may also cause cognitive enhancement (82). The exact mechanism remains uncertain. It is proposed that targeting different stimulation sites such as the precuneus, parietal region, or cerebellum could improve executive dysfunction in the future.

In conclusion, this meta included 20 RCTs of executive function in patients with VCI treated by rTMS. It not only provides comprehensive evidence of its efficacy but also has some limitations: (1) the RCT has a range of subjective scales and no objective indications, which increases the heterogeneity of indicators; (2) there is less literature in foreign languages (7 foreign papers of 20 papers in this meta-analysis), which is probably related to the absence of rTMS in the latest guidelines for post-stroke cognitive dysfunction (83). So there is a greater need for further studies to promote the application of the study.

## 5 Conclusion

rTMS is effective in the treatment of the executive function of VCI. Higher frequency, lower intensity, iTBS, combined comprehensive therapies, and longer interventions are more effective. There are some limitations in this study, and further exploration requires more objective metrics and optimal parameters to enhance its application.

## Data availability statement

The original contributions presented in the study are included in the article/supplementary material, further inquiries can be directed to the corresponding author.

## Author contributions

XW: Data curation, Formal analysis, Methodology, Software, Writing – original draft. QD: Conceptualization, Software, Writing – review & editing. YuL: Resources, Supervision, Writing – review & editing. TL: Resources, Supervision, Writing – review & editing. YaL: Data curation, Investigation, Writing – review & editing. JY: Investigation, Project administration, Writing – review & editing. WZ: Funding acquisition, Project administration, Writing – review & editing.

## Footnotes

^1^http://www.stata.com
